# Geo-classification of drug-resistant travel-associated *Plasmodium falciparum* using *Pfs47* and *Pfcpmp* gene sequences (USA, 2018–2021)

**DOI:** 10.1128/aac.01203-24

**Published:** 2024-11-12

**Authors:** Edwin Pierre-Louis, Julia Kelley, Dhruviben Patel, Christina Carlson, Eldin Talundzic, David Jacobson, Joel Leonard Nicholas Barratt

**Affiliations:** 1 Laboratory Science and Diagnostics Branch, Division of Parasitic Diseases and Malaria, National Center for Emerging and Zoonotic Infectious Diseases, Centers for Disease Control and Prevention, Atlanta, Georgia, USA; 2 Oak Ridge Institute for Science and Education, Oak Ridge, Tennessee, USA; 3 Williams Consulting LLC, Atlanta, Georgia, USA; The Children's Hospital of Philadelphia, Philadelphia, Pennsylvania, USA

**Keywords:** *Plasmodium falciparum*, malaria, *Pfs47*, *Pfcpmp*, next-generation sequencing, molecular surveillance, drug resistance, travel history, geographic prediction

## Abstract

Travel-related malaria is regularly encountered in the United States, and the U.S. Centers for Disease Control and Prevention (CDC) characterizes *Plasmodium falciparum* drug-resistance genotypes routinely for travel-related cases. An important aspect of antimalarial drug resistance is understanding its geographic distribution. However, specimens submitted to CDC laboratories may have missing, incomplete, or inaccurate travel data. To complement genotyping for drug-resistance markers *Pfcrt*, *Pfmdr1*, *Pfk13*, *Pfdhps*, *Pfdhfr*, and *PfcytB* at CDC, amplicons of *Pfs47* and *Pfcpmp* are also sequenced as markers of geographic origin. Here, a bi-allele likelihood (BALK) classifier was trained using *Pfs47* and *Pfcpmp* sequences from published *P. falciparum* genomes of known geographic origin to classify clinical genotypes to a continent. Among *P. falciparum*-positive blood samples received at CDC for drug-resistance genotyping from 2018 to 2021 (*n* = 380), 240 included a travel history with the submission materials, though 6 were excluded due to low sequence quality. Classifications obtained for the remaining 234 were compared to their travel histories. Classification results were over 96% congruent with reported travel for clinical samples, and with collection sites for field isolates. Among travel-related samples, only two incongruent results occurred; a specimen submitted citing Costa Rican travel classified to Africa, and a specimen with travel referencing Sierra Leone classified to Asia. Subsequently, the classifier was applied to specimens with unreported travel histories (*n* = 140; 5 were excluded due to low sequence quality). For the remaining 135 samples, geographic classification data were paired with results generated using CDC’s Malaria Resistance Surveillance (MaRS) protocol, which detects single-nucleotide polymorphisms in and generates haplotypes for *Pfcrt*, *Pfmdr1*, *Pfk13*, *Pfdhps*, *Pfdhfr*, and *PfcytB*. Given the importance of understanding the geographic distribution of antimalarial drug resistance, this work will complement domestic surveillance efforts by expanding knowledge on the geographic origin of drug-resistant *P. falciparum* entering the USA.

## INTRODUCTION

Parasites of the genus *Plasmodium* are the causative agents of malaria, which is transmitted in the bite of infected female *Anopheles* mosquitoes (1). Five *Plasmodium* species infect humans: *P. falciparum*, *P. vivax*, *P. ovale*, *P. knowlesi*, and *P. malariae* (2), with *P. falciparum* constituting the greatest threat to global health (3). Approximately half the world’s population is at risk of malaria, and in 2022, an estimated 249 million malaria cases occurred worldwide, resulting in an estimated 608,000 fatalities (3). Sub-Saharan Africa (AF) bears the highest burden of malaria compared to other endemic regions, and about 76% of malaria-related deaths occur among children under 5 years old (3). Despite significant progress in global malaria control efforts in recent decades, a major impediment to sustained malaria control has been emerging antimalarial drug resistance.

The World Health Organization (WHO) recommends artemisinin-based combination therapies (ACTs) to treat uncomplicated *Plasmodium* infections (4). Six first-line ACTs are available (i.e., artemether-lumefantrine, artenusate-amodiaquine, artesunate-mefloquine, artesunate plus sulfadoxine-pyrimethamine, dihydroartemisinin-piperaquine, and artesunate-pyronaridine) (4, 5). For infections caused by susceptible parasites, ACTs rapidly reduce parasitemia, while decreased parasite clearance may be linked to mutations in the Kelch 13 gene (*Pfk13*) responsible for artemisinin partial resistance (6–8). Chloroquine (CQ) is not recommended for use in South America, Asia, and Africa, where CQ resistance—associated with mutations in the chloroquine resistance transport (*Pfcrt*) gene—is common (9, 10). Resistance to CQ has not been widespread in Central America historically, though recent patterns of migration in this region heighten the risk of CQ resistance spreading there (11–13). Drug resistance-conferring mutations also occur in other *P. falciparum* genes, including cytochrome b (*PfcytB*: atovaquone) (14), dihydrofolate reductase (*Pfdhfr*: pyrimethamine, proguanil, and cycloguanil) (15–18), dihydropteroate synthase (*Pfdhps*: sulfadoxine) (19), and multidrug-resistant protein 1 (*Pfmdr1*: chloroquine, piperaquine, artesunate, mefloquine, and lumefantrine, among others) (20, 21). Drug-resistance profiles vary by geography and may spread between regions under permitting circumstances (22, 23). While the European Union and United States are non-endemic for *P. falciparum* malaria, imported cases are frequently encountered (24–26). Approximately 2,000 imported malaria infections are reported to US public health authorities annually, most occurring in US residents who traveled to places where malaria transmission occurs (27, 28).

To examine drug-resistance genotypes among U.S. travel-related *P. falciparum* infections, the Division of Parasitic Diseases and Malaria (DPDM) at the U.S. Centers for Disease Control and Prevention (CDC) developed the MaRS protocol, which screens for antimalarial drug-resistance genotypes among travel-related *P. falciparum* cases (26). The MaRS approach uses targeted deep amplicon sequencing (TADS) to detect single-nucleotide polymorphisms (SNPs) across six *P. falciparum* drug-resistance markers; *Pfcrt*, *Pfmdr1*, *Pfk13*, *Pfdhps*, *Pfdhfr*, and *PfcytB* (29–31). In addition to the six drug-resistance genes, the conserved *Plasmodium* membrane protein (*Pfcpmp*) gene and sexual-stage surface protein 47 (*Pfs47*) gene are sequenced as markers of geographic origin (32, 33).

Here, different SNP combinations in *Pfs47* and *Pfcpmp* were explored for their utility in classifying *P. falciparum* to a continent. Six classification models were constructed by training a previously described bi-allele likelihood (BALK) classifier (34) on *Pfs47* and *Pfcpmp* sequences extracted from >10,000 publicly available *P. falciparum* genomes (MalariaGEN) (35) of known geographic origin. While as few as two *Pfs47* SNP sites are reportedly sufficient to assign *P. falciparum* to a continent of origin (33), the classification performance of more complex models was explored here (totalling six *Pfs47* and *Pfcpmp* SNP combinations). Classification accuracy was evaluated for each model by comparing the geographic origin of 10 sets of 627 MalariaGEN “test” genomes from Africa (*n* = 267), Asia-Oceania (*n* = 338), and Central/South America (CSA) (*n* = 22, not well represented in MalariaGEN) to classifications obtained for the same genomes, noting that the test genomes had been excluded from the 10 data sets used to train each model.

US travel-associated *P. falciparum*-positive samples submitted to CDC from 2018 to 2021 (*n* = 380) were sequenced at *Pfs47* and *Pfcpmp*, plus *Pfcrt*, *Pfmdr1*, *Pfk13*, *Pfdhps*, *Pfdhfr*, and *PfcytB* using the MaRS protocol. A BALK classification model was selected from among the six described above based on their performance when evaluated against MalariaGEN data. The chosen model was subsequently applied to data sequenced from 369 *P*. *falciparum*-positive bloods including 135 specimens for which travel data were not supplied to the laboratory upon sample submission, noting that 11 of the 380 samples had insufficient sequencing coverage at *Pfs47* and *Pfcpmp* for analysis.

## MATERIALS AND METHODS

### Samples

U.S. State Public Health Laboratories may receive whole-blood samples from patients diagnosed with malaria by local healthcare providers within their jurisdictions. A subset of these malaria-positive blood samples are voluntarily transferred to CDC for malaria molecular surveillance. Travel information is requested for the malaria-positive samples submitted for molecular surveillance purposes but was not always provided. In these cases, the travel data were considered missing, and no attempt was made to follow up on missing data within the scope of this report. The travel data that were provided on sample submission forms did not undergo additional verification within the scope of this analysis and may therefore be incomplete or inaccurate and should be viewed as preliminary. Whole-blood samples are processed at CDC for downstream molecular testing/genotyping. From 2018 to 2021, CDC received 514 presumptive malaria parasite positive samples for downstream processing.

### DNA extraction and *Plasmodium* confirmation

DNA was isolated from whole-blood samples (*n* = 514) using the QIAmp DNA blood Mini kit following the whole-blood protocol (Qiagen, CA, USA), with elution in 150 µL of buffer AE and reapplication of the eluate to the same spin column, followed by re-spinning, to maximize DNA isolation. The presence of *Plasmodium* DNA in these extracts was confirmed using three previously described photo-induced electron transfer (PET)-PCR assays that provide species-level confirmation for the presence of *P. falciparum*, *P. malariae*, *P. ovale*, and/or *P. vivax* (36). All PCR runs were accompanied by a negative template control and one or more positive template controls. Only samples positive for *P. falciparum* (*n* = 380) were subjected to downstream analysis here.

### TADS

The 380 *P*. *falciparum*-positive samples were subjected to TADS using a previously described protocol (26), targeting full-length genes encoding the *Pfcrt*, *Pfmdr1*, *Pfk13*, *Pfdhps*, *Pfdhfr*, and *PfcytB* proteins, which are recognized by the WHO as determinants of antimalarial drug resistance (12). *Pfs47* and *Pfcpmp* gene sequences were also sequenced via TADS as predictors of geographic origin. These eight loci were amplified in a separate single-plex reaction each using Phusion high-fidelity DNA polymerase (New England BioLabs, USA) and *P. falciparum*-specific primers targeting these genes (Table S1) (26). Following PCR, amplicon clean-up and normalization were performed using a SequalPrep Normalization Plate Kit (Thermo Fisher Scientific, USA). Library preparation was performed using a Nextera XT v2 library preparation kit (Illumina, USA). Paired-end sequencing was performed on the Illumina MiSeq platform using a MiSeq Reagent Nano 500-cycle v2 kit (Illumina). Multiple sequencing runs were required to sequence all eight markers for the 380 samples. At least one *P. falciparum* reference strain was included in each library for quality control purposes. Reference strains sequenced in this study included the wild-type (susceptible) strains 3D7 and D6; two *Pfmdr1* mutant strains, HB3 and 7G8; and strains Dd2, FC27, MRA1236, and W2 (File S1; Table S2). To rule out cross-contamination between samples during library preparation, all libraries included at least one negative water sample that was processed alongside positive samples at all steps.

### Data processing (CDC-sequenced samples)

#### Drug-resistance SNP detection

For antimalarial drug-resistance SNP detection, a published workflow described elsewhere (37) was used. Briefly, paired-end reads were imported into Geneious Prime (https://www.geneious.com) for adapter-trimming and quality-filtering using BBDuk (quality score >35, minimum length = 50) (38). Bowtie2 (default parameters) was used to map reads to susceptible/wild-type 3D7 reference sequences of *Pfcrt*, *Pfmdr1*, *Pfk13*, *Pfdhps*, *Pfdhfr*, and *PfcytB* (GenBank accession numbers/gene IDs: 2655199, 813045, 814205, 2655294, 9221804, and LR605957.1: PF3D7_MIT02300, respectively). A variant was called if it achieved at least fivefold read depth and an allele frequency of at least 5%. Custom Jupyter code (see reference [37] – 03_geneious_reports directory) was used to simplify Geneious output annotations and create graphs. A variant allele frequency (VAF) of >95% was considered a mutant genotype, while a VAF of <95% was considered a mixed genotype. Amino acid haplotypes for each drug-resistance gene were collated after extracting variant calls at SNP loci recognized as determinants of drug resistance (22). Mixed genotypes—evidence of multiclonal infections (26, 39–42)—were excluded from subsequent analyses.

#### Geographic prediction: variant calling

Quality trimmed reads (quality score >30 and minimum length = 50 in BBDuk) were aligned to the GRCh38 human reference genome (GenBank: GCA_000001405.29) using Bowtie2 (43), and samtools (39) was used to extract reads not mapping to the human genome. Remaining reads were mapped to *Pfs47* and *Pfcpmp* reference sequences (GenBank gene ID: 814213 & 813178) using Bowtie2. Aligned reads were merged and sorted using samtools. Variants were called using the GATK4 (41, 42) pipeline with default parameters (File S2).

#### Geographic prediction: model evaluation

A previously described BALK classification approach (34) was trained to predict a parasite’s geographic origin using *Pfs47* and *Pfcpmp* sequences. First, bi-allelic SNPs from *Pfs47* and *Pfcpmp* were selected as variant sites for classifier training and subsequent classification. For *Pfs47*, 83 SNPs described by Molina-Cruz et al. (33) served as a starting point (before applying later filtering steps). For *Pfcpmp*, we used bcftools (39) to identify high-quality SNPs from samples in the MalariaGEN Pf7 database (35) using the following parameters: only include bi-allelic SNPs with a “PASS” variant call filter, and only retain SNPs with a variant quality score log odds greater than 6. This yielded 61 SNPs within *Pfcpmp* (File S1; Table S3). Next, variant calls at each SNP locus were extracted from each sample (*n* = 20,865) within the MalariaGEN Pf7 variant call files using bcftools. An additional filtering step was applied to remove *Pfs47* SNPs that were not bi-allelic in the Pf7 database, resulting in a final set of 69 *Pfs47* SNPs (File S1; Table S3). The BALK classifier was trained using six SNP combinations (six classification panels) to optimize classification performance (File S1; Table S3):

1. **Panel 1:** all *Pfs47* and *Pfcpmp* SNPs (69 & 61 SNPs, respectively).
2. **Panel 2:** only the 69 *Pfs47* SNPs.
3. **Panel 3:** only the 61 *Pfcpmp* SNPs.
4. **Panel 4:** only two *Pfs47* SNPs (Pf3D7_13:1879488; Pf3D7_13:1879470), described previously as being informative for continent-level classification (33).
5. **Panel 5:** all *Pfs47* SNPs but excluding the 2 highly informative SNPs (67 SNPs).
6. **Panel 6:** all *Pfs47* & *Pfcpmp* SNPs excluding the 2 informative *Pfs47* SNPs (128 SNPs).

The analysis plan in online supplemental File S3 (Fig. S1) was used to assess classification performance when trained on each panel. Briefly, samples not passing the quality control steps described in MalariaGEN (35) were filtered out, and SNP calls were transformed into a barcode for each sample (File S3; Fig. S1). Samples with missing data at one or more loci were removed. Due to differences in genotype completeness (panel dependent), variable numbers of samples remained in each classification panel this step: Panel 1 (*n* = 11,005); Panel 2 (*n* = 14,891); Panel 3 (*n* = 11,436); Panel 4 (*n* = 15,996); Panel 5 (*n* = 14,899); and Panel 6 (*n* = 11,011). A list of all samples included in each panel is available in File S1 and Table S4.

To evaluate classifier performance, 627 samples were randomly selected from 30 countries (File S1; Table S5), from those remaining in the filtered MalariaGEN Pf7 database for each of the six panels. These would serve as a test data set (File S2). Samples remaining after removal of the 627 test samples served as training data for each classification model. Random subsampling of 627 test samples (File S1; Table S5) was repeated to create 10 training and test data sets for each of the six panels (File S3; Fig. S1). Classification models were trained using all 10 subsampling iterations of the six panels, using the “train” function of the BALK software. Training was performed at three levels of geographic resolution—country, region, and continent—to assess performance at each level. After training, test data sets were supplied to each of the six models (10 iterations each) using the “predict” function of the BALK software. BALK software outputs include the top three classification categories for each sample, scored between 0 and 1, with higher scores indicating higher confidence in the classification category provided. Classification performance was evaluated using Matthew’s correlation coefficient (MCC) (File S2) (44) with Kruskal-Wallis and Dunn tests used to determine if the distribution of MCC scores were significantly different between the six panels.

#### Geographic prediction: analyzing CDC-sequenced samples

A classification panel was selected from among those obtaining the highest MCC values for subsequent classifications, where 1 of the 10 iterations was randomly selected. The chosen model was applied to samples and reference strains sequenced at CDC, using genotypes constructed via the GATK4 pipeline (see above) as input. Samples missing data at 25% or more of the SNPs targeted by the selected classification model were excluded.

## RESULTS

### Malaria samples received by CDC

Of the 514 presumptive malaria positive samples received within the study period, 380 were *P. falciparum*-positive by PET-PCR (36) (Table 1). Of these 380 samples, travel information was submitted to the CDC laboratory for 243 specimens, predominantly citing travel to Africa. PET-PCR confirmed mixed species infections comprising the mixes Pf and Po, Pf and Po, Pf and Po, and Pf and Pv for four samples from patients reporting travel to African countries (File S1; Table S6: samples 18GNNVxxx0136PfB1230_S51, 18GNNVxxx0137PfB1230, 18WAPAxxA0138PfB1230_S53, and 18CIPAxxP0139PfB1230, respectively).

**TABLE 1:** Summary of samples received by CDC as part of domestic malaria surveillance

Parameter	Year				Total
	2018	2019	2020	2021	
Number of samples submitted to CDC					
Samples received^*a*^	157	193	45	119	514
*P. falciparum* positive	103	157	35	85	380
*P. falciparum* positive with travel information provided^*b*^	75	103	19	46	243
Travel history (continent) for 380 *P. falciparum* positives					
Africa	74	103	17	46	240
Asia/Oceania	1	0	0	0	1
Central/South America	0	0	2	0	2
Unknown	28	54	16	39	137

^*a*^ Includes samples submitted for suspicion of *P. falciparum* malaria infection; 380 of 514 were *P. falciparum*- positive by PET-PCR.

^*b*^ The subset of these 380 *P. falciparum* positives accompanied by a reported travel history.

### Performance of *Pfcpmp* and *Pfs47* SNP panels

**Fig 1:**
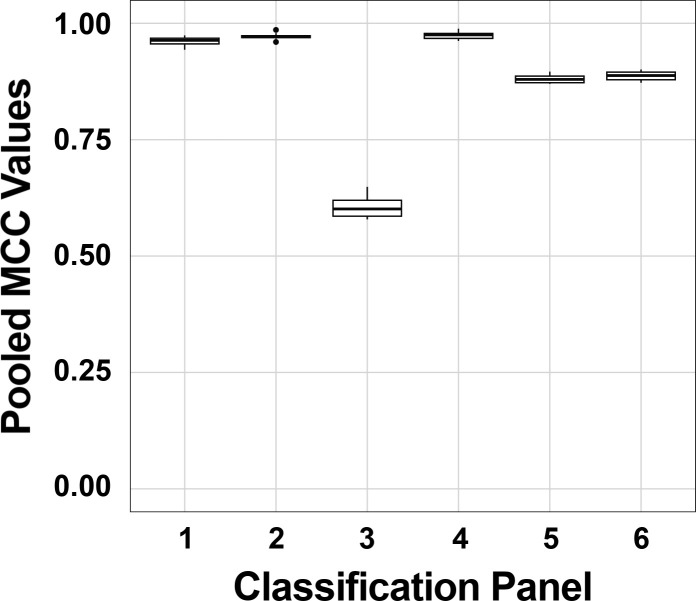
Pooled MCC values for six continent-level classification panels applied to the MalariaGEN Pf7 database. Mean pooled MCC values for panels 1, 2, and 4 exceeded 0.96 and were not significantly different from each other. Models where the two highly informative *Pfs47* SNPs were excluded (panels 3, 5, and 6) performed significantly worse than panels where these two SNPs were present (panels 1, 2, and 4). Panel 1 was selected for downstream analyses. Panel 1: all *Pfs47* and *Pfcpmp* SNPs (69 and 61 SNPs, respectively); Panel 2: only the 69 *Pfs47* SNPs; Panel 3: only the 61 *Pfcpmp* SNPs; Panel 4: only 2 *Pfs47* SNPs described previously as being informative for continent-level classification; Panel 5: all *Pfs47* SNPs but excluding the 2 highly informative SNPs (67 SNPs); Panel 6: all *Pfs47* and *Pfcpmp* SNPs excluding the 2 informative *Pfs47* SNPs (128 SNPs).

Regardless of the panel, region (regions defined by MalariaGEN [35]) and country-level classifications were unreliable (File S3; Fig. S2). Thus, only continent-level classifications were considered, including AF, CSA, and Asia/Oceania (AO). The model trained using two *Pfs47* SNPs alone (Panel 4) (33) was most accurate (mean pooled MCC = 0.974, Fig. 1), though its performance was not significantly greater than the *Pfs47* and *Pfcpmp* combined approach (Panel 1, mean pooled MCC = 0.962, *P* value = 0.213) nor using all SNPs from the *Pfs47* gene (Panel 2, mean pooled MCC = 0.972, *P* value = 0.778). When using the model trained on all *Pfs47* SNPs alone but excluding the two informative SNPs (Panel 5), and the model using all *Pfs47* and *Pfcpmp* SNPs but excluding the two informative *Pfs47* SNPs (Panel 6), accuracy remained high, though a drop in performance was observed (Panel 5: MCC = 0.880; Panel 6: MCC = 0.888) (Fig. 1). Supported by these data, the model trained on Panel 1 (all 69 *Pfs47* SNPs and 61 *Pfcpmp* SNPs) was selected for subsequent classifications.

### Geographic classification of clinical *P. falciparum* samples

Sequence data were generated for 380 *P. falciparum*-positive clinical samples plus 33 reference strains. Eleven clinical samples and one reference strain had poor coverage at more than 25% of SNP sites in Panel 1 and were excluded, leaving 369 clinical samples and 32 reference strains (File S1; Table S6). Classification accuracy was first evaluated for samples with a reported travel history (*n* = 234), as well as sequenced reference strains (*n* = 32). Assuming accurate travel histories, performance was high (>0.95 MCC, Table 2; File S1; Table S6), similar to MCC values obtained using the MalariaGEN data set (Fig. 1, Panel 1). Only two clinical cases received incongruent classifications; a specimen with information suggesting travel in Sierra Leone obtained an AO classification, and one specimen with reported travel to Costa Rica received an AF classification. The genotype for the specimen discordant for Sierra Leone travel displayed heterozygosity at the Pf3D7_13_v3:1879488 SNP (File S1; Table S6), the only heterozygous allele detected at this SNP site among samples sequenced at CDC. This sample had a C to T transition at Pf3D7_13_v3:1879488, and the variant allele frequency was 0.682, suggesting a multiclonal infection. Among samples in the Panel 1 MalariaGEN training and test data sets (*n* = 11,005), 14 were heterozygous at this SNP site, and all 14 originated from Bangladesh or India. African samples sequenced at CDC and those in the Panel 1 MalariaGEN Pf7 test/training data set were homozygous for cytosine, and all Asian and Central/South American samples were homozygous for thymine at the Pf3D7_13_v3:1879488 locus. The incongruent Costa Rica travel genotype also had “Africa-specific” cytosines at the two informative *Pfs47* SNPs (33) (Pf3D7_13:1879488 and Pf3D7_13:1879470, File S1; Table S6). For the 140 samples without travel histories, 135 passed the inclusion criteria for subsequent classification: 134 classified to AF, one to CSA, and 0 to AO (File S1; Table S6). Notably, all samples obtained a prediction value of 1 (i.e., maximum confidence). Overall, of the 369 CDC-sequenced samples with a geographic prediction, 365 were classified to AF, 2 to AO, and 2 to CSA (File S1; Table S6).

**TABLE 2:** Classification accuracy for clinical samples (Cl.) & control (Co.) strains sequenced at CDC^*a*^

Origin	True positives^*b*^		True negatives^*b*^		False positives^*b*^		False negatives^*b*^		MCC for classification, Panel 1^*c*^		
	Cl.	Co.	Cl.	Co.	Cl.	Co.	Cl.	Co.	Cl.	Co.	Combined
AF	230	10	2	22	1	0	1	0	0.662	1.00	0.956
CSA	1	12	232	20	0	0	1	0	0.706	1.00	0.962
AO	1	10	232	22	1	0	0	0	0.706	1.00	0.956
Total	232	32	466	64	2	0	2	0	0.987	1.00	0.989

^*a*^ MCC values are lower for clinical columns because the sample sizes for Central/South America (CSA, *n* = 2) and Asia/Oceania (AO, *n* = 1) are small, reflecting sampling bias toward Africa (AF). Combining controls from these regions with clinical samples provides a truer representation of classifier performance. See File S2 and Table S2 for a full description of the reference strains sequenced.

^*b*^ For these calculations, true-positive, true-negative, false-positive, and false-negative categories were applied under the assumption that the travel histories were accurate despite being unverified and preliminary (see Materials and Methods).

^*c*^ Scores based on comparisons to reported travel histories (or known origins for control strains).

### Drug-resistance mutations

Sufficient sequencing quality was obtained for the six drug-resistance genes at most SNPs within 380 clinical samples, though depth varied by gene (Files S2 and S3; Fig. S3). Mutations in *Pfdhfr* conferring resistance to pyrimethamine were present in 365 (96.1%) of the samples (Table 3). Mutations in *Pfdhps* and *Pfmdr* associated with sulfadoxine and chloroquine resistance were present in 371 (97.6%) and 226 (59.5%) samples, respectively. For 213 of 380 samples, mutations associated with chloroquine resistance were detected in *Pfcrt*. Artemisinin resistance-associated *Pfk13* alleles were found in three samples. Drug resistance-conferring mutations were not detected in *cytochrome b*, a marker associated with atovaquone-proguanil resistance. Malaria drug-resistance profiles were next examined by continent, as determined by BALK classification.

**TABLE 3:** Samples with mutations at six drug-resistance genes^*a*^^,^^*b*^

	2018	2019	2020	2021	Total
*Pfdhps*	102 (99%)	155 (99%)	34 (97%)	80 (94%)	371 (98%)
*Pfdhfr*	101 (98%)	149 (95%)	33 (94%)	82 (96%)	365 (96%)
*Pfcrt*	54 (52%)	121 (77%)	11 (31%)	27 (32%)	213 (56%)
*Pfmdr1*	62 (60%)	66 (42%)	22 (63%)	76 (89%)	226 (59%)
*PfcytB*	0 (0%)	0 (0%)	0 (0%)	0 (0%)	0 (0%)
*Pfk13*	1 (1%)	1 (1%)	0 (0%)	1 (1%)	3 (0.8%)
Total sequenced^*c*^	103	157	35	85	380^*d*^

^*a*^ Number of samples with at least one resistance-associated mutation associated by year.

^*b*^ Counts and other details for each specific mutation can be found in Files S1 and S2.

^*c*^ The denominator for each year.

^*d*^ Total number of samples sequenced at each gene across all years.

#### Drug-resistance haplotypes among AF-classified US clinical samples

**Fig 2:**
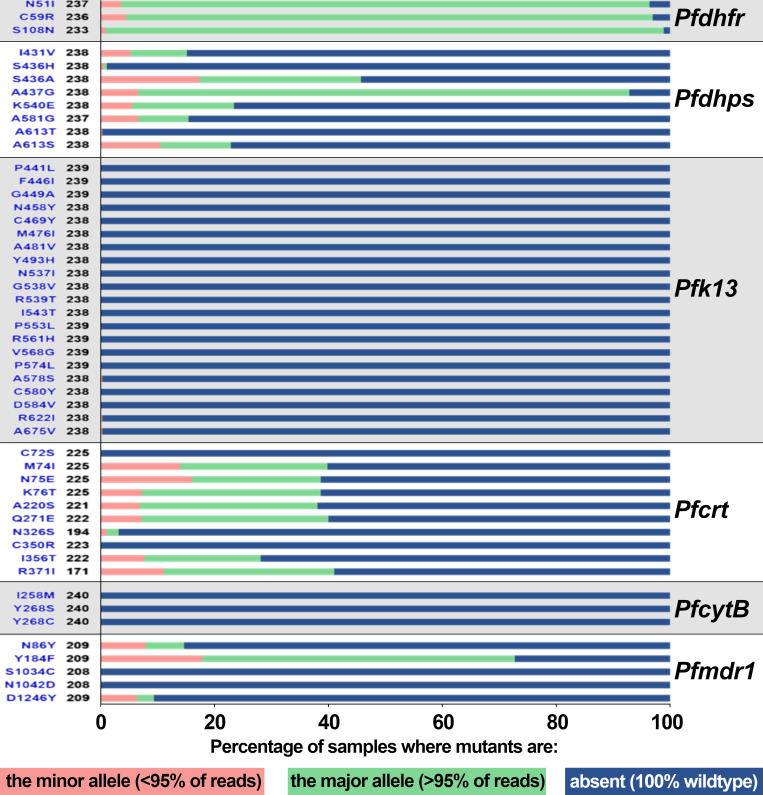
Frequencies of 49 non-synonymous drug-resistance SNPs among AF-classified US samples. Associated non-synonymous SNPs in malaria drug-resistance genes are shown. The graph depicts the frequencies of the wild-type allele, and alleles (with frequency ≥95% and <95%) associated with drug-resistance SNPs. SNPs are listed as gene name: wild-type amino acid-codon position-mutant amino acid (left) and total number of samples (right) on the *y*-axis. Allele frequencies are represented as percentages on the *x*-axis. The *Pfk13* A578S polymorphism is not associated with a resistance phenotype (26). Blue text on the left side of the panel indicates the non-synonymous mutation, while black text refers to the number of AF-classified samples for which the mutation obtained sufficient coverage for SNP calling. Refer to File S1 and Table S7 for complete details on mutation frequencies. Specifically, the figure includes *Pfdhps* mutations associated with sulfadoxine resistance, *Pfdhfr* mutations with pyrimethamine and proguanil resistance, *Pfcytb* mutations with atovaquone resistance, *Pfmdr1* and *Pfcrt* mutations with chloroquine resistance, and *Pfk13* mutations associated with artemisinin partial resistance. The genotype frequencies depicted in this figure are discussed in detail in File S2. Note that this figure lists all non-synonymous mutations that are recognized as conferring antimalarial drug resistance (regardless of geographic origin) for each drug-resistant marker examined (left column, blue text). All subsequent discussions on the specific haplotypes identified in this study reference these codons.

**Fig 3:**
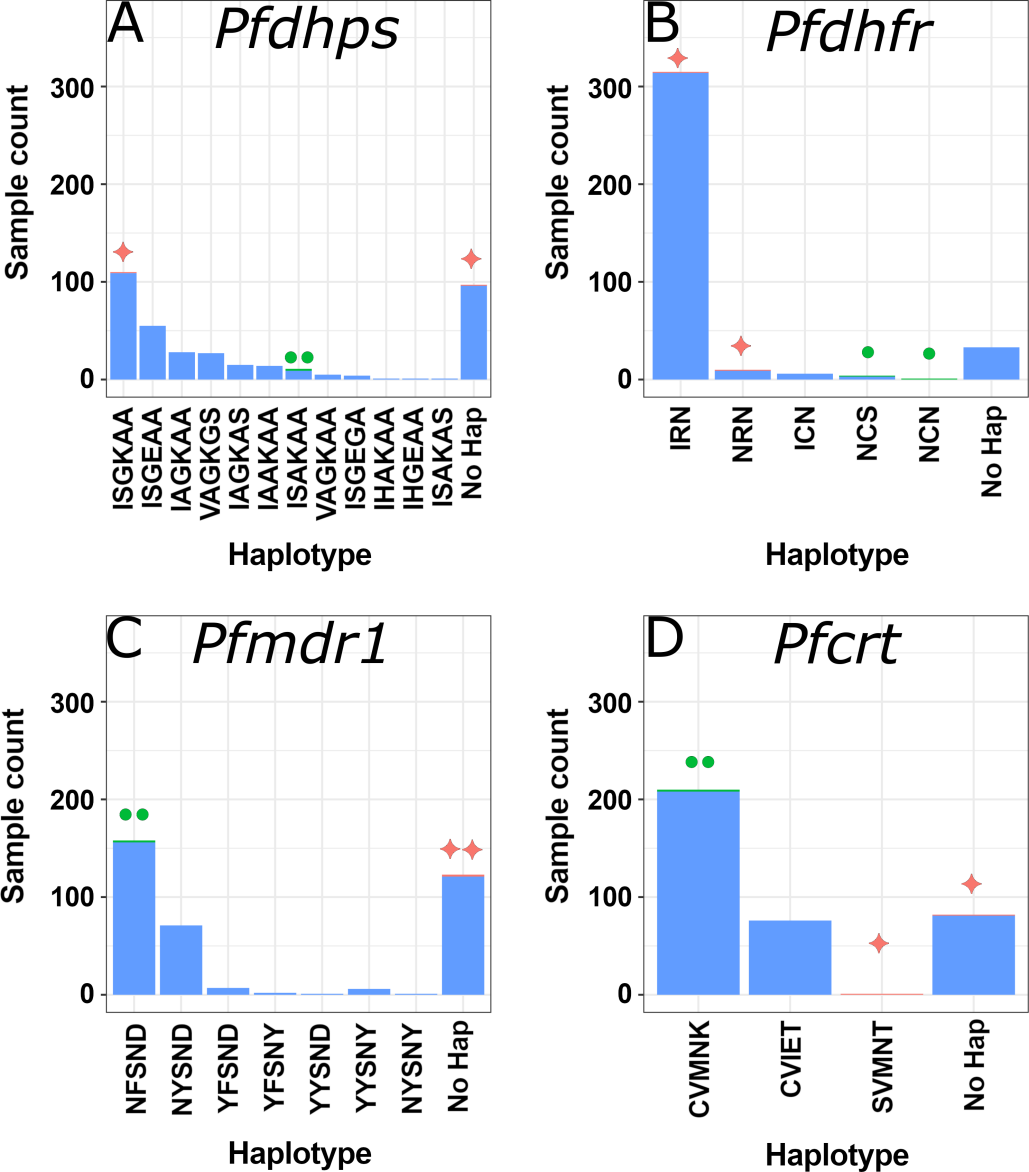
Drug-resistance haplotype distribution for *Pfdhps*, *Pfdhfr*, *Pfmdr1*, and *Pfcrt*. The total number of samples with specified drug resistance-associated haplotypes identified in (**A**) *Pfdhps*, (**B**) *Pfdhfr*, (**C**) *Pfmdr1*, and (**D**) *Pfcrt*. The 365 AF-classified samples represent the bulk of each. Bars with one or two pink diamonds (✦) contain one or two samples classified to AO, respectively. Bars with one or two green circles (●) represent those including one or two CSA-classified samples, respectively. The number of samples with either a mixed allele haplotype or lacking sufficient coverage for an SNP call are included in the “no hap” bars. *Pfk13* and *PfcytB* are not shown as all samples possessed the wild-type allele or were represented in the no hap bars. Refer to File S1 and Table S7 for complete details on mutation frequencies.

Drug-resistance haplotype analyses utilized samples for which the major allele was either the mutant allele or wild type (i.e., excluding complex mixes). For details on SNP percentages, refer to Fig. 2; File S1; Table S7. Twelve *Pfdhps* haplotypes were detected, including the ISAKAA wild type. Of the 365 AF-classified samples, 96 were mixed or lacked coverage for at least one of six variable *Pfdhps* amino acids. Among the AF-classified samples remaining (*n* = 269), the most abundant *Pfdhps* haplotypes were ISGKAA (*n* = 109, 41%), ISGEAA (*n* = 55, 21%), IAGKAA (*n* = 28, 11%), and VAGKGS (*n* = 27, 10%). Remaining haplotypes were found at low abundance including the ISAKAA wild type (nine clinical samples, 3%) (Fig. 3; File S1; Tables S7 and S8).

Roughly 9% (*n* = 33) of AF-classified samples had an uncalled haplotype in *Pfdhfr,* leaving 332 for analysis. Nearly 95% (*n* = 314) of remaining samples had the triple mutant IRN, while NRN, ICN, and the wild-type NCS haplotype were found in less than 10 samples each (Fig. 3; File S1; Table S8). The CVMNK wild type was the most common haplotype detected in *Pfcrt*, with 208 of 284 (73%) samples without a mixed genotype carrying this combination. The triple mutant CVIET was found in 76 samples (27%). The *Pfcrt* mutant SVMNT (45) was not detected in AF-classified samples (Fig. 3; File S1; Table S8). For *Pfmdr1*, the single mutant NFSND (*n* = 156, 65%) and the wild type NYSND (*n* = 71, 29%) made up over 93% of haplotypes in AF-classified samples without a mixed genotype (*n* = 244) (Fig. 3). Five additional low-abundance *Pfmdr1* haplotypes were detected (Fig. 3; File S1; Table S8). Finally, excluding samples with mixed haplotypes which could not be definitively resolved, we found only wild-type haplotypes in *Pfk13* (YRIC) and *PfcytB* (IY). Notably, the discordant clinical sample obtaining an AF classification possessed mixed haplotypes for *Pfdhps* and *Pfmdr1* but carried the IRN triple mutant at *Pfdhfr* and CVIET at *Pfcrt.* The specific codons referenced for all genes are provided in File S1 and Table S8. These are also discussed in File S2.

#### Haplotype frequencies among US samples classified to CSA and AO

Both CSA-classified samples carried the *Pfdphs* ISAKAA wild-type haplotype, the *Pfmdr1* NFSND single mutant, the *Pfcrt* CVMNK wild type, and the *Pfk13* YRIC wild type. For *Pfdhfr,* the NCN haplotype was found in one CSA sample (Fig. 3; File S1; Table S8), and the NCS wild ype was found in the other. Neither sample had a categorizable *PfcytB* haplotype. The two AO-classified samples had wild-type *Pfk13* and *PfcytB* haplotypes though different *Pfdhfr* haplotypes: the AO-classified discordant specimen, IRN, and India travel specimen, NRN (Fig. 3; File S1; Table S8). *Pfmdr1* alleles could not be called for the AO-classified discordant specimen, which had mixed *Pfdhps* and *Pfcrt* genotypes. The India travel sample had the ISGKAA *Pfdhps* haplotype and was the only sample among the CDC clinical samples to have the SVMNT *Pfcrt* haplotype (Fig. 3; File S1; Table S8).

## DISCUSSION

The *P. falciparum* geographic classification model described, based on *Pfcpmp* and *Pfs47* (Panel 1), represents a useful tool for characterizing the geographic origin of drug-resistant *P. falciparum* strains to the continent level, giving a more complete picture on the origin of drug-resistant *P. falciparum* genotypes entering the United States. Among the six *Pfs47* and *Pfcpmp* SNPs combinations investigated for BALK model training, Panel 1 (capturing all 130 SNPs examined) was selected. There are two reasons for this. First, while the *Pfs47* two-SNP approach (Panel 4) obtained the highest MCC values, relying solely on two SNPs risks excluding samples when insufficient coverage is observed for one or both of these SNPs. This exclusion would be unnecessary for samples that might simultaneously obtain sufficient coverage for many of the other informative SNPs considered in Panel 1. Second, the performance of Panel 1 was not significantly different from Panel 4. Thus, Panel 1 performs well while also being robust to fluctuations in coverage commonly obtained across amplicons. Using Panel 1, continent-level classifications were largely congruent for reference strains and clinical samples (MCC >0.98). Panel 1 also performed well when applied to reference strains of known origins (AF, CSA, and AO) sequenced here. This was important to demonstrate classification accuracy for data sequenced at CDC across all three *P. falciparum-*endemic continents, given the bias toward African samples in the present clinical data set. Our data also indicate that *Pfcpmp* represents a diverse marker but does not perform well as a marker of geographic origin on its own. However, the results observed for Panel 6 suggest that *Pfcpmp* may offer some complimentary benefit when sequenced in conjunction with *PfS47*.

Discordant classifications occurred for 2 of 234 cases with a reported travel history. Notably, travel histories were extracted from sample submission forms in the present study without any additional verification. As a nationally notifiable disease, malaria epidemiological data are also submitted to CDC via the National Malaria Surveillance System (NMSS) and the National Notifiable Diseases Surveillance System (NNDSS). NMSS and NNDSS epidemiological data are subjected to thorough verification via subsequent public health investigations. Verification of travel histories provided on sample submission forms was not performed within the scope of this study, so the travel data presented here should be viewed as unverified and preliminary and may be incomplete or inaccurate. Discordance between these preliminary travel histories and the geographic classifications is expected for a low number of clinical specimens. For situations where laboratory specimens can be linked to an epidemiological investigation, discordances like this may prompt clarification from further investigation procedures. It is important to consider that geographic classifications of travel-associated malaria parasites are subject to limitations; travelers are mobile and may experience multiple exposures in areas of malaria transmission. Therefore, sequences derived from travel-associated malaria parasites were not used to train the BALK classifier in the present study. Instead, genetic data from field isolates obtained from the MalariaGEN Pf7 database were used to build a classification model that was useful to identify genetic signatures that link a travel-associated parasites’ genotype to a most likely continent of origin. The cytosine/thymine heterozygosity observed in one discordant sample at Pf3D7_13_v3:1879488, which is one of two key SNPs important for *Pfs47* continent level prediction, led the classifier to assign this sample to AO. Heterozygosity at this locus is unusual, as all other samples sequenced here were homozygous at this SNP; AF-classified samples all carried cytosine, and all AO and CSA samples and reference strains had a thymine. Less than 0.13% of samples in the Panel 1 data sets from the MalariaGEN Pf7 database release were heterozygous at this site, and samples that were heterozygous originated solely from South Asia. Future work is needed to ascertain the prevalence of heterozygous alleles at these two *Pfs47* SNP loci from a more diverse sample set to identify underlining trends influencing heterozygosity in *Pfs47*. The other discordant sample classified to AF with strong support as the *Pfs47* cytosine-cytosine combination at Pf3D7_13:1879488 and Pf3D7_13:1879470 reportedly only occurs in Africa (33).

Pyrimethamine-resistant and sulfadoxine-resistant parasites were common in AF-classified samples. Mutations in three *Pfdhfr* codons (N51I, C59R, and S108N) are associated with pyrimethamine resistance, with IRN triple mutants being highly resistant (18, 46). Over 94% of AF-classified samples carried the IRN mutant, and single or double mutants were detected in 5.4% of these samples. Less than 1% of African genotypes were of the *Pfdhfr* NCS wild type, supporting prior reports of triple *Pfdhfr* mutants being widespread across Africa (17, 46). Interestingly, the AF-classified discordant case and the AO-classified discordant case both carried the IRN triple mutant. Sulfadoxine resistance is linked to mutations in *Pfdhps*, and roughly 5% of samples sequenced here were wild type, the same proportion of *Pfdhps* wild-type genotypes reported previously among US cases (26). Similarly, the two most common *Pfdhps* haplotypes (ISGKAA and ISGEAA) comprised approximately 60% of samples in the present and in prior (26) domestic data sets.

Chloroquine-resistant genotypes were detected in both *Pfcrt* and *Pfmdr1* among cases with an African travel history. CVIET was the most common *Pfcrt* mutant, an expected result as CVIET is the most common chloroquine resistance mutation in Africa, and more than 95% of clinical samples sequenced here were genetically classified to Africa. The SVMNT double mutant, conferring resistance to chloroquine and amodiaquine (47, 48), was present in one sample, one with a laboratory-reported history of travel to India that was classified to AO. This SVMNT mutant has been detected in Africa, South America, and Asia, though its prevalence in Africa is low (48). Most *Pfmdr1* genotypes comprised the Y184F single mutations (i.e., NFSND), which confers chloroquine resistance, though to a limited effect (49). More robust chloroquine-resistant phenotypes are associated with other codons in *Pfmdr1*, particularly N86Y (49). Mutations at codon 86 were found in less than 6% of African genotypes here, in agreement with previous work indicating that the N86Y mutation is less common in Africa than the Y184F mutation and the wild type (26, 50). It is worth noting that for many *P. falciparum* strains, resistance to lumefantrine and mefloquine is mediated by *pfmdr1* copy number variations (CNVs) (51), which are difficult to detect by amplicon sequencing and represents a notable limitation of the current amplicon sequencing approach that could be addressed by employing alternative sequencing methodologies. Similarly, piperaquine resistance is mediated by plasmepsin II and/or III gene duplications (52), genes not captured by the MaRS amplicon sequencing protocol. For US domestic malaria drug-resistance screening, whole-genome sequencing could help address CNV detection and would facilitate retrospective re-analysis of sequenced samples should novel mechanisms of antimalarial drug resistance be identified in the future.

Mutations in *Pfk13* and *PfcytB* confer resistance to artemisinins and malarone (atovaquone-proguanil [AP]), respectively (6–8, 14). *Pfk13* variants were detected in only three African samples here. While these *Pfk13* mutations represent minor variants, this is nonetheless a notable finding, particularly the R662I and A675V mutations (53). These mutations were once confined to Asia, and based on recently described timelines for the emergence of artemisinin partial resistance in Africa (53), these 2018 samples from the present study may be among the earliest detections of these mutations on the continent. Given that the estimated prevalence of validated non-synonymous mutations for *Pfk13* in India is 2% (54) and that the single case with Asian travel history reported travel to India, the absence of *Pfk13*-related drug-resistant variants is unsurprising for this sample. In the USA and Europe, AP is the most commonly prescribed treatment for uncomplicated malaria (55, 56). Tracking drug resistance in returning travelers is important for maintaining informed medical guidelines. No samples carried atovaquone-resistant *PfcytB* mutations, reflecting results from a previous US data set, where 1 of 234 travel-linked cases possessed a drug-resistant genotype (Y268S) (26). Indeed, cytochrome B mutations that confer drug resistance are reportedly rare (57), supporting that AP remains an efficacious malaria treatment.

There may be some value in using the geographic distribution of drug-resistance haplotypes to inform a sample’s geographic classification when Panel 1 yields a confounding result, as for the two discordantly classified specimens. However, it should be noted that few drug-resistance haplotypes have a clear geographic distribution, and such haplotypes may shift in geographic profile over time due to selection via widespread use of similar antimalarial drug treatment regimens. Furthermore, the AO-classified discordant specimen did not have callable haplotypes at three of the six resistance markers, partially due to the presence of complex genotypes. While the mixed or uncalled alleles for this AO-classified specimen may indicate a complex infection potentially related to a true Asian origin, between 20% and 30% of our AF-classified samples had uncalled haplotypes at these genes, casting doubt on the utility of using mixed genotypes for classification via the current methods. It seems plausible, however, that an approach based on a larger panel of markers analyzed using alternate models or algorithms could alleviate this challenge in the future.

### Conclusions

While Panel 1 yielded informative continent-level classifications, an expanded panel including additional markers could provide more granular classifications. For example, a panel for geographically classifying *P. vivax* captures 113 SNPs across numerous genomic regions provides subcontinent-level classifications within AO (e.g., Western Asia, Western Southeast Asia, Eastern Southeast Asia, and Oceania) (34). Sequence-based geographic classification of human-infecting *Plasmodium* has been a recent research focus of great interest (58–63). However, outstanding questions remain, such as whether specific panels are applicable in regions not sampled/evaluated during a panel’s development or whether markers providing resolution within a narrow region (e.g., a country) remain efficacious in other regions. The biological function of the markers utilized for classification—particularly *PfS47*—is also an important consideration that might impact the classifier’s performance in the future. Specifically, certain mutations within *PfS47* are subject to immunoselection with the vector, with different vector species driving the selection of specific mutations (64, 65). Thus, classification efficacy is linked to the geographic distribution of different *Plasmodium* vector species and could be influenced by changes in vector distribution. As alluded to above, utilizing an expanded panel of markers (i.e., possessing diverse biological functions) may help alleviate this, in addition to regularly updating the training data set to include strains very recently isolated in the field and continually evaluating the concordance between classifications and reported travel histories.

Whole-genome sequencing and SNP-barcoding approaches provide valuable insights into *P. falciparum* geographic trends (59–62) and, when viewed in aggregate, complement our understanding of the epidemiology of travel-related malaria in the USA, including our knowledge of antimalarial drug-resistance trends among US travel-patient cohorts. Similar geographic classification analyses have also been used to complement epidemiological investigations of autochthonous *P. vivax* malaria cases occurring in the USA in 2023. A more granular classification approach than the one described here for *P. falciparum* might provide data that are actionable from a broader global health perspective. For example, among the US travel-patient cohort, malaria infections are acquired from diverse locations, which may facilitate detection of novel antimalarial drug-resistance mutations in an area or expansion of known drug-resistance determinants into new areas. This might prove informative particularly within certain African subregions where ongoing field surveillance of antimalarial drug resistance is lacking.

One relatively unexplored aspect of *P. falciparum* drug resistance includes analysis of genomic regions flanking drug-resistance genes to ascertain if specific drug-resistance mutations arose independently in different populations or if they originated in one population and spread to others via a recent selective sweep (66–68). Such analyses could provide vital clues on how and where mutations arise and where they are spreading; this knowledge could improve the effectiveness of public health interventions aiming to limit the spread of resistance. Examining the six drug-resistance genes studied here also remains an important aspect of malaria surveillance. Finally, around 2,000 cases of travel-related malaria are reported annually in the USA, yet only 5%–10% of samples are submitted to CDC for genotyping (28). Sequencing a larger proportion of travel-associated US malaria genotypes for drug-resistance screening and geographic classification could support US malaria preparedness, particularly in light of recent reports of autochthonous US *P. falciparum* malaria transmission (69).

## Data Availability

All data are publicly available through the National Center for Biotechnology Information (NCBI) BioProject number PRJNA428490, under the accession numbers: SRR9184251–SRR9184407, SRR27503149–SRR27503425, and SRR28459326–SRR28459566.

## SUPPLEMENTAL MATERIAL

File S1 aac.01203-24-s0001.xlsx Primer sequences, sample metadata, classification results, drug resistance genotypes, and other information.

File S2 aac.01203-24-s0002.docx Supplemental methods and results.

File S3 aac.01203-24-s0003.pdf Figures S1 to S3.

ASM does not own the copyrights to Supplemental Material that may be linked to, or accessed through, an article. The authors have granted ASM a non-exclusive, world-wide license to publish the Supplemental Material files. Please contact the corresponding author directly for reuse.
