# Supplementary material for: Geo-classification of drug-resistant travel-associated Plasmodium falciparum using Pfs47 and Pfcpmp gene sequences (USA, 2018–2021)
Source: Antimicrob Agents Chemother. 2024 Nov 12;68(12):e01203-24. doi: 10.1128/aac.01203-24 (PMC11619247; doi:10.1128/aac.01203-24)
Supplement: File S2 — Supplemental methods and results. [file aac.01203-24-s0002.docx]

**Supplementary Text**

**Geo-classification of drug-resistant travel-associated *Plasmodium falciparum* using *Pfs47* and *Pfcpmp* gene sequences (USA, 2018-2021).**

Edwin Pierre-Louis^1,2†^, Julia Kelley^1†^, Dhruviben Patel^1,3^, Christina Carlson^1^, Eldin Talundzic^1^, David Jacobson^1^, Joel Leonard Nicholas Barratt^1*^

^1^Laboratory Science and Diagnostics Branch, Division of Parasitic Diseases and Malaria, National Center for Emerging and Zoonotic Infectious Diseases, Centers for Disease Control and Prevention, Atlanta, GA, USA.

^2^Oak Ridge Institute for Science and Education, TN, USA.

^3^Williams Consulting LLC, Atlanta, GA, USA.

Running Head: Geographic classification of drug resistance genotypes for US malaria cases, 2018-2021

^*^Address correspondence to Joel Barratt (JB: [nsk9@cdc.gov](mailto:nsk9@cdc.gov))

Key Words: *Plasmodium falciparum*, Malaria, *Pfs47*, *Pfcpmp*, Next Generation Sequencing, Molecular Surveillance, Drug Resistance, Travel History, and Geographic Prediction

^†^Edwin Pierre-Louis and Julia Kelly contributed equally to this work.

# Table of Contents

[Table of Contents 2](#_Toc174005726)

[Supplementary Methods – Bioinformatics 3](#_Toc174005727)

[Variant Calling Pipeline 3](#_Toc174005728)

[Determining Best Performing Classification Panel 3](#_Toc174005729)

[Selection of 627 Samples for Test Dataset 3](#_Toc174005730)

[Calculating Matthew’s Correlation Coefficient for Classification Panels 4](#_Toc174005731)

[Matthew’s Correlation Coefficient (MCC) for MalariaGEN samples: 4](#_Toc174005732)

[Matthew’s Correlation Coefficient for CDC sequenced samples: 5](#_Toc174005733)

[Supplementary Results 5](#_Toc174005734)

[Read Depth for SNPs Associated with Antimalarial-Resistance. 5](#_Toc174005735)

[Genotype Frequencies for Malaria Drug Resistance among U.S. Isolates Classified to Africa 6](#_Toc174005736)

[Genotype frequencies for malaria drug resistance among U.S. isolates classified to Central / South America and Asia / Oceania 8](#_Toc174005737)

[Table S1. Breakdown of mutations observed in the single isolates classified to Central / South America (CSA) and Asia-Oceania (A-O) 8](#_Toc174005738)

[References 9](#_Toc174005739)

# Supplementary Methods – Bioinformatics

## Variant Calling Pipeline

CleanSam was used to soft-clip alignments extending beyond the reference and then Genomic Variant Call Format (GVCF) files were generated for each sample using HaplotypeCaller (1, 2). GVCFs were combined and genotyped with GATK4 CombineGVCFs and GenotypeGVCFs (with the –include-non-variant-sites flag), respectively (1, 2). Genotypes were reformatted downstream for geographic prediction using GATK4 VariantsToTable and a custom python script, which extracts the SNP sites of interest and denotes SNP calls as ‘N’ for heterozygous and ‘X’ for missing data.

## Determining Best Performing Classification Panel

### Selection of 627 Samples for Test Dataset

Between 2 and 60 sequenced samples (i.e., *P. falciparum* field isolates) per country (mean samples per country = 20.9) were randomly subsampled from the the MalariaGEN Pf7 database to create the Test Dataset for each iteration of each classification panel. The number of samples per country always matched the distribution listed in Supplemental Table S5. We chose an uneven number of samples per country to mimic a potential ‘real’ surveillance dataset where there will typically be an uneven distribution of samples per country/region. The countries with the fewest number of samples in the Test Dataset sample distribution were also the countries that had a smaller number of samples that passed the overall inclusion criteria. This was done for model building reasons, as it is not feasible to remove more than 20% of all available samples for a country from the model building dataset and place them in the Test Dataset, since we still need to retain a diversity of samples per country to build an informative model. For example, there were only 21 samples from Peru (4 selected for Test Dataset), 21 samples from Ethiopia (3 selected for Test Dataset), 24 samples from Madagascar (3 selected for Test Dataset), 34 samples from Mozambique (4 selected for Test Dataset), 55 samples from Gabon (6 selected for Test Dataset) and 57 samples from Burkina Faso (9 selected for Test Dataset). Ultimately, only continent level predictions were used and thus such attention to country-level sample representation may have not been necessary because we do not use country level predictions; however, the approach we outlined here could be useful when conducting a similar analysis on a SNP dataset that can prediction country or region of origin.

### Calculating Matthew’s Correlation Coefficient for Classification Panels

The BALK classifier (3) provides the three most likely answers for each sample’s geographic prediction. A probability between 0 and 1 is also reported, with 1 being highest confidence that the sample originates from the reported answer, and the sum of probabilities equal to 1. An example of the output for a single sample is below. We used the top prediction as the predicted geographic origin when calculating Matthew’s Correlation Coefficient.

| **Sample** | **Continent 1** | **Probability 1** | **Continent 2** | **Probability 2** | **Continent 3** | **Probability 3** |
| --- | --- | --- | --- | --- | --- | --- |
| Test 1 | Africa | 0.95 | Asia / Oceania | 0.04 | Central / South America | 0.01 |

### Matthew’s Correlation Coefficient (MCC) for MalariaGEN samples:

We used the ‘Country’ column from the MalariaGEN Pf7 data release (4) to determine the sample’s country of origin, we used the MalariaGEN ‘Population’ column to determine the region, and we assigned each sample to a continent based on their country of origin. We used the geographic metadata from the Pf7 data release as the ‘true’ geographic origin and compared our predicted geographic values to the ‘true’ value to determine the number of true positives, false positives, true negatives, and false negatives. We then used the ‘mcc’ tool within the ‘mltools’ (5) library in R to calculate MCC for each country/region/continent. We then took the total number of true positives, false positives, true negatives, and false negatives across all geographic regions to calculate a ‘pooled’ MCC, which represents the overall performance at each level of geographic region.

### Matthew’s Correlation Coefficient for CDC sequenced samples:

We used the same approach as described as above for CDC sequenced samples to calculate MCC. For reference strains cultured in lab, we used the reported origin of each sample based on a literature search (Supplemental Table S1). For clinical samples, we used the laboratory reported travel histories that were provided to CDC on standardized sample submission forms alongside physical samples.

# Supplementary Results

## Read Depth for SNPs Associated with Antimalarial-Resistance.

The levels of read depth varied for different drug resistance-associated polymorphisms in six genes (*Pfcrt*, *Pfmdr1*, *Pfk13*, *Pfdhps*, *Pfdhfr*, and *PfcytB*). The statistics for read were as follows: median, 129.5; mean, 218.5; 1^st^ quartile, 50; 3^rd^ quartile, 291; minimum, 2; and maximum, 3244. Notably, the read depth was not consistent across the full-length genes including the mitochondrial genome. Lower read depths were observed for *Pfcrt* and *Pfmdr1* polymorphisms compared to those for *Pfk13*, *Pfhps*, *Pfdhfr*, and the *PfcytB* (Supplemental Figure S3).

## Genotype Frequencies for Malaria Drug Resistance among U.S. Isolates Classified to Africa

Among the 49 drug-resistance related and reportable SNPs studied across the 365 Africa-predicted samples (6), 51% (25/49) accounted for minor mutant alleles, defined as those with frequencies < 95%, and 42% (21/49) were major mutant alleles, defined as those with frequencies ≥ 95% (Figure 2). Our genotyping analysis revealed the prevalence of 8 amino acid variants in the *Pfdhps* gene: I431V in 55 of 363 (15%), S436A in 166 of 363 (46%), S436H in 4 of 363 (1%), A437G in 337 of 363 (93%), K540E in 85 of 363 (23%), A581G in 56 of 363 (15%), A613S in 83 of 363 (23%), A613T in 1 of 363 (0%) (Figure 2 and Supplemental File S1 - Tab 7). While seven of the eight amino acid variations were observed previously in a study employing the MaRS protocol (7), I431V was added to the current domestic survey as it is considered a reportable codon of interest (8-10). The I431V, S436A, S436H, A437G, K540E, A581G, A613S, and A613T polymorphisms were found as both major and minor alleles and were respectively distributed in 36 (10%), 103 (28%), 2 (1%), 313 (86%), 65 (18%), 32 (9%), 45(12%), and 0 (0%) of samples as major alleles, and as minor alleles in 19 (5%), 63 (17%), 2 (1%), 24 (7%), 20 (6%), 24 (7%), 38 (11%), and 1 (0%) samples. In contrast, mutations were much more common at 3 polymorphisms in the *Pfdhfr* gene (N51I, C59R and S108N), which were detected in 96% (347/360), 97% (349/360) and 99% (352/356) of samples, respectively (Figure 2; Supplemental File S1 - Tab 7). Across these samples, 93% of N51I, 93% of C59R and 98% of S108N were found as major alleles whereas 4% of N51I, 4% of C59R and 1% of S108N were classified as minor alleles.

Among these 365 African-classified samples, polymorphisms in the *Pfcrt* gene correspondingly accounted for 2-33% of major and 1-16 % of minor alleles (Figure 2 and Supplemental File S1 - Tab 7). The mutations M74I, N75E, K76T, A220S, Q271E, N326S, I356T, R371I were found in 89 (26%), 78 (23%), 108 (31%), 105 (31%), 110 (33%), 6 (2%), 69 (20%), 75 (30%) samples, respectively, as major alleles while M74I, N75E, N326S, K76T, A220S, Q271E, I356T, R371I were observed in minor alleles in 48 (14%), 55 (16%), 3 (1%), 25 (7%), 23 (7%), 24 (7%), 26 (8%), 28 (11%) samples as minor alleles.

In the *Pfmdr1* gene, N86Y, Y184F, S1034C, N1042D and D1246Y polymorphisms were found in 22 (7%), 181 (55%), 0 (0%), 0 (0%) and 10 (3%) samples as major alleles and in 26 (8%), 59 (18%), 0 (0%), 0 (0%) and 21 (6%) samples as minor alleles. Together, N86Y was found in 48 of 328 (15%), Y184F in 240 of 330 (73%), S1034C in 0 of 328 (0%), N1042D in 0 of 329 (0%) and D1246Y 31 of 331 (9%) samples (Fig. 3 and Supplemental File S1 - Tab 7). Furthermore, in the *Pfk13* gene, only 3 polymorphisms -- A578S, A675V and R622I -- were found. All three SNPs were found in 0 samples as major allele and in 1 sample each as a minor allele (Figure 2 and Supplemental File S1 - Tab 7). Notably, these specimens were introduced to the U.S. from patients reporting travel to Kenya (A578S), Sudan (A675V) and Nigeria (R622I). Finally, there was no evidence of polymorphism in the *PfcytB* gene marker amongst the reportable SNPs of interest, namely I258M and Y268S, which are SNPs that have previously been linked to AP resistance (11, 12). It is worth noting that AP is commonly administered to treat uncomplicated *P. falciparum* malaria in travelers in the U.S. (13, 14).

## Genotype frequencies for malaria drug resistance among U.S. isolates classified to Central / South America and Asia / Oceania

Four samples were classified as having a non-African origin (Table S1). One sample originated in India with SNPs identified in the following markers only: *Pfcrt* (C72S, K76T and A220S), *Pfdhps* (A437G) and *Pfdhfr* (C59R, S108N). The *Pfcrt* alleles in this specimen have been reported from Asia previously (15). Another sample with laboratory-reported travel history to Sierra Leone classified to Asia / Oceania and its SNPs distribution for the markers was as follows: *Pfcrt* (M74I, N75E, K76T, A220S, Q271E, I356T ‘MIX’, and R371I), *Pfdhps* (A437G, K540E ‘MIX’) and *Pfdhfr* (N51I, C59R, and S108N). In the two samples with a Central / South American classification, one of the samples had no travel history provided while the other had travel reported as Dominican Republic. Two markers detected in the sample with travel reported as Dominican Republic had one mutation in each of *Pfmdr1* (Y184F) and *Pfdhfr* (S108N), while the *Pfmdr1* Y184F SNP was the only mutation identified from the sample with the unreported travel history and a classification to Central /South America.

### Table S1. Breakdown of mutations observed in the single isolates classified to Central / South America (CSA) and Asia-Oceania (A-O)

|  | **Isolates with Central / South America classification** | | **Isolates with Asia / Oceania classification** | |
| --- | --- | --- | --- | --- |
| **Laboratory Reported Travel History** | **Dominican Republic** | **Missing** | **India** | **Sierra Leone** |
| *Pfdhps* | WT | WT | A437G | A437G, K540E (Mix) |
| *Pfdhfr* | S108N | WT | C59R, S108N | N51I, C59R, S108N |
| *Pfcrt* | WT | WT | C72S, K76T, A220S | M74I, N75E, K76T, A220S, Q271E, I356T (Mix), R371I, |
| *Pfmdr1* | Y184F | Y184F | No coverage | No coverage |
| *PfcytB* | WT | WT | WT | WT |
| *Pfk13* | WT | WT | WT | WT |

# References

1. McKenna A, Hanna M, Banks E, Sivachenko A, Cibulskis K, Kernytsky A, et al. The Genome Analysis Toolkit: a MapReduce framework for analyzing next-generation DNA sequencing data. Genome research. 2010;20(9):1297-303.

2. Van der Auwera GA, O'Connor BD. Genomics in the cloud: using Docker, GATK, and WDL in Terra: O'Reilly Media; 2020.

3. Trimarsanto H, Amato R, Pearson RD, Sutanto E, Noviyanti R, Trianty L, et al. A molecular barcode and web-based data analysis tool to identify imported Plasmodium vivax malaria. Communications biology. 2022;5(1):1411.

4. Hamid MMA, Abdelraheem MH, Acheampong DO, Ahouidi A, Ali M, Almagro-Garcia J, et al. Pf7: an open dataset of Plasmodium falciparum genome variation in 20,000 worldwide samples. Wellcome open research. 2023;8.

5. Gorman B. mltools: Exploratory and diagnostic machine learning tools for R. <https://github.com/ben519/mltools>: GitHub; 2018.

6. Organization WH. Report on antimalarial drug efficacy, resistance and response: 10 years of surveillance (2010-2019): World Health Organization; 2020.

7. Talundzic E, Ravishankar S, Kelley J, Patel D, Plucinski M, Schmedes S, et al. Next-generation sequencing and bioinformatics protocol for malaria drug resistance marker surveillance. Antimicrobial Agents and Chemotherapy. 2018;62(4):10.1128/aac. 02474-17.

8. Oguike MC, Falade CO, Shu E, Enato IG, Watila I, Baba ES, et al. Molecular determinants of sulfadoxine-pyrimethamine resistance in Plasmodium falciparum in Nigeria and the regional emergence of dhps 431V. International Journal for Parasitology: Drugs and Drug Resistance. 2016;6(3):220-9.

9. Sutherland CJ, Fifer H, Pearce RJ, bin Reza F, Nicholas M, Haustein T, et al. Novel pfdhps haplotypes among imported cases of Plasmodium falciparum malaria in the United Kingdom. Antimicrobial agents and chemotherapy. 2009;53(8):3405-10.

10. Zhao L, Pi L, Qin Y, Lu Y, Zeng W, Xiang Z, et al. Widespread resistance mutations to sulfadoxine-pyrimethamine in malaria parasites imported to China from Central and Western Africa. International Journal for Parasitology: Drugs and Drug Resistance. 2020;12:1-6.

11. Kessl JJ, Ha KH, Merritt AK, Lange BB, Hill P, Meunier B, et al. Cytochrome b mutations that modify the ubiquinol-binding pocket of the cytochrome bc1 complex and confer anti-malarial drug resistance in Saccharomyces cerevisiae. Journal of Biological Chemistry. 2005;280(17):17142-8.

12. Plucinski MM, Huber CS, Akinyi S, Dalton W, Eschete M, Grady K, et al., editors. Novel mutation in cytochrome B of Plasmodium falciparum in one of two atovaquone-proguanil treatment failures in travelers returning from same site in Nigeria. Open forum infectious diseases; 2014: Oxford University Press.

13. Huang Z, Tatem AJ. Global malaria connectivity through air travel. Malaria journal. 2013;12:1-11.

14. LaRocque RC, Rao SR, Lee J, Ansdell V, Yates JA, Schwartz BS, et al. Global TravEpiNet: a national consortium of clinics providing care to international travelers—analysis of demographic characteristics, travel destinations, and pretravel healthcare of high-risk US international travelers, 2009–2011. Clinical infectious diseases. 2012;54(4):455-62.

15. Awasthi G, Prasad G, Das A. Population genetic analyses of Plasmodium falciparum chloroquine receptor transporter gene haplotypes reveal the evolutionary history of chloroquine-resistant malaria in India. International journal for parasitology. 2011;41(7):705-9.
