# Supplementary material for: Geo-classification of drug-resistant travel-associated Plasmodium falciparum using Pfs47 and Pfcpmp gene sequences (USA, 2018–2021)
Source: Antimicrob Agents Chemother. 2024 Nov 12;68(12):e01203-24. doi: 10.1128/aac.01203-24 (PMC11619247; doi:10.1128/aac.01203-24)
Supplement: File S3 — Figures S1 to S3. [file aac.01203-24-s0003.pdf]

Figure S1

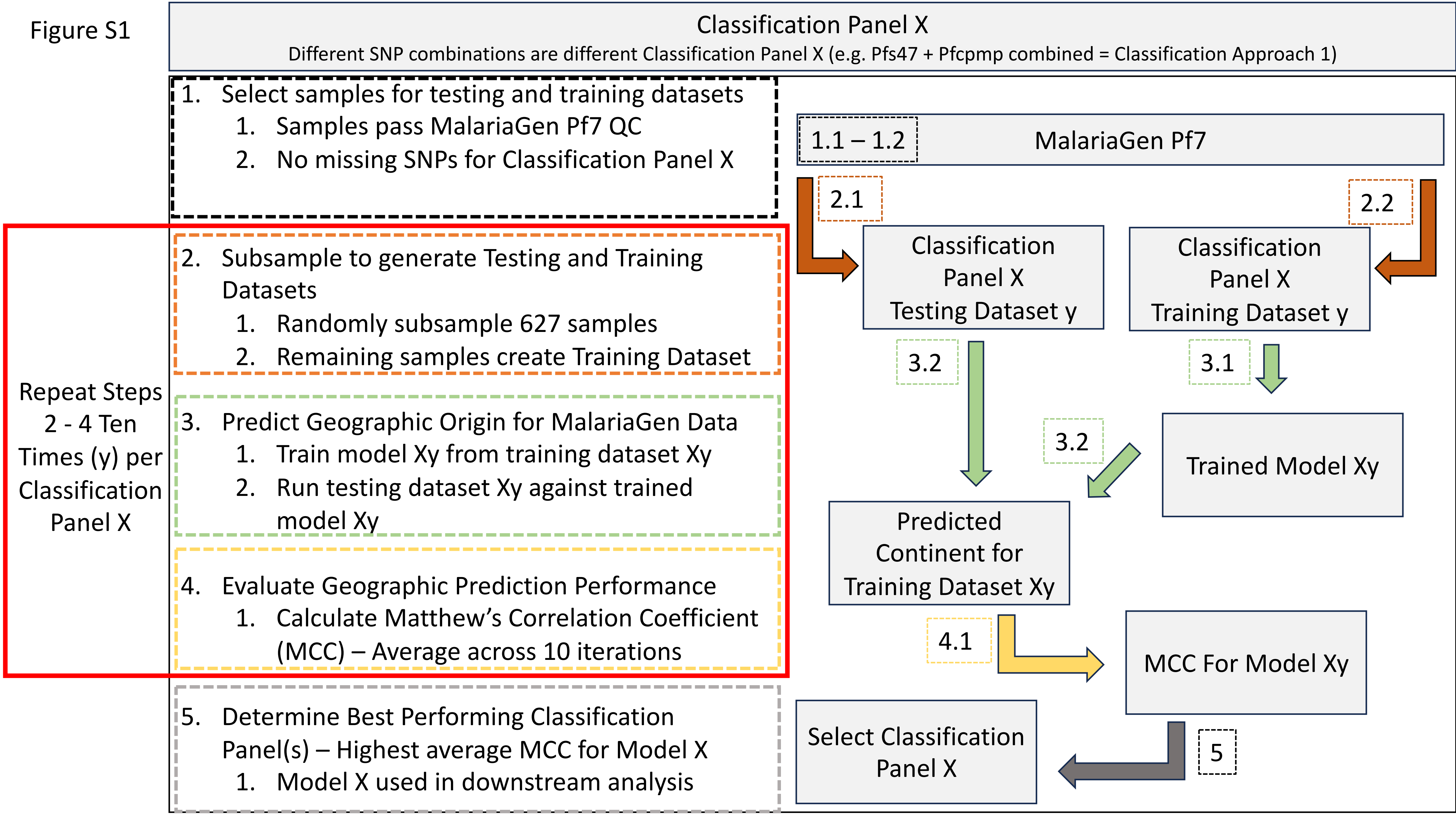

Classification Panel X
Different SNP combinations are different Classification Panel X (e.g. Pfs47 + Pfcpmp combined = Classification Approach 1)
1. Select samples for testing and training datasets
1. Samples pass MalariaGen Pf7 QC
2. No missing SNPs for Classification Panel X
Repeat Steps 2 - 4 Ten Times (y) per Classification Panel X
2. Subsample to generate Testing and Training Datasets
1. Randomly subsample 627 samples
2. Remaining samples create Training Dataset
3. Predict Geographic Origin for MalariaGen Data
1. Train model Xy from training dataset Xy
2. Run testing dataset Xy against trained model Xy
4. Evaluate Geographic Prediction Performance
1. Calculate Matthew's Correlation Coefficient (MCC) – Average across 10 iterations
5. Determine Best Performing Classification Panel(s) – Highest average MCC for Model X
1. Model X used in downstream analysis
1.1 – 1.2
MalariaGen Pf7
2.1
2.2
Classification Panel X Testing Dataset y
Classification Panel X Training Dataset y
3.2
3.1
3.2
Trained Model Xy
Predicted Continent for Training Dataset Xy
4.1
MCC For Model Xy
5
Select Classification Panel X

Figure S1 Legend:

Analysis workflow for determining which Classification Panel yields the highest MCC values. We started with the complete MalariaGen Pf7 dataset and filtered out samples missing data at any of the SNPs listed for each Classification Panel (Supplemental File S1 - Tab 3). We randomly sampled 627 samples from 30 countries to build a testing and training dataset, then trained a model from the training dataset for each Classification Panel. We ran the test dataset against the trained model for each respective Classification Panel and compared geographic prediction to sample origin (as listed in the MalariaGen Pf7 dataset). We repeated this process 10 times for each classification panel

Classification Panel 1: All Pfs47 and Pfcpmp SNPs (69 & 61 SNPs, respectively),

Classification Panel 2: Only the 69 Pfs47 SNPs,

Classification Panel 3: Only the 61 Pfcpmp SNPs,

Classification Panel 4: Only two Pfs47 SNPs (Pf3D7_13:1879488; Pf3D7_13:1879470), described as being highly informative (29) ,

Classification Panel 5: All Pfs47 SNPs but the two highly informative SNPs are excluded (67 SNPs),

Classification Panel 6: All Pfs47 & Pfcpmp SNPs excluding the two highly informative Pfs47 SNPs (128 SNPs).

Figure S2

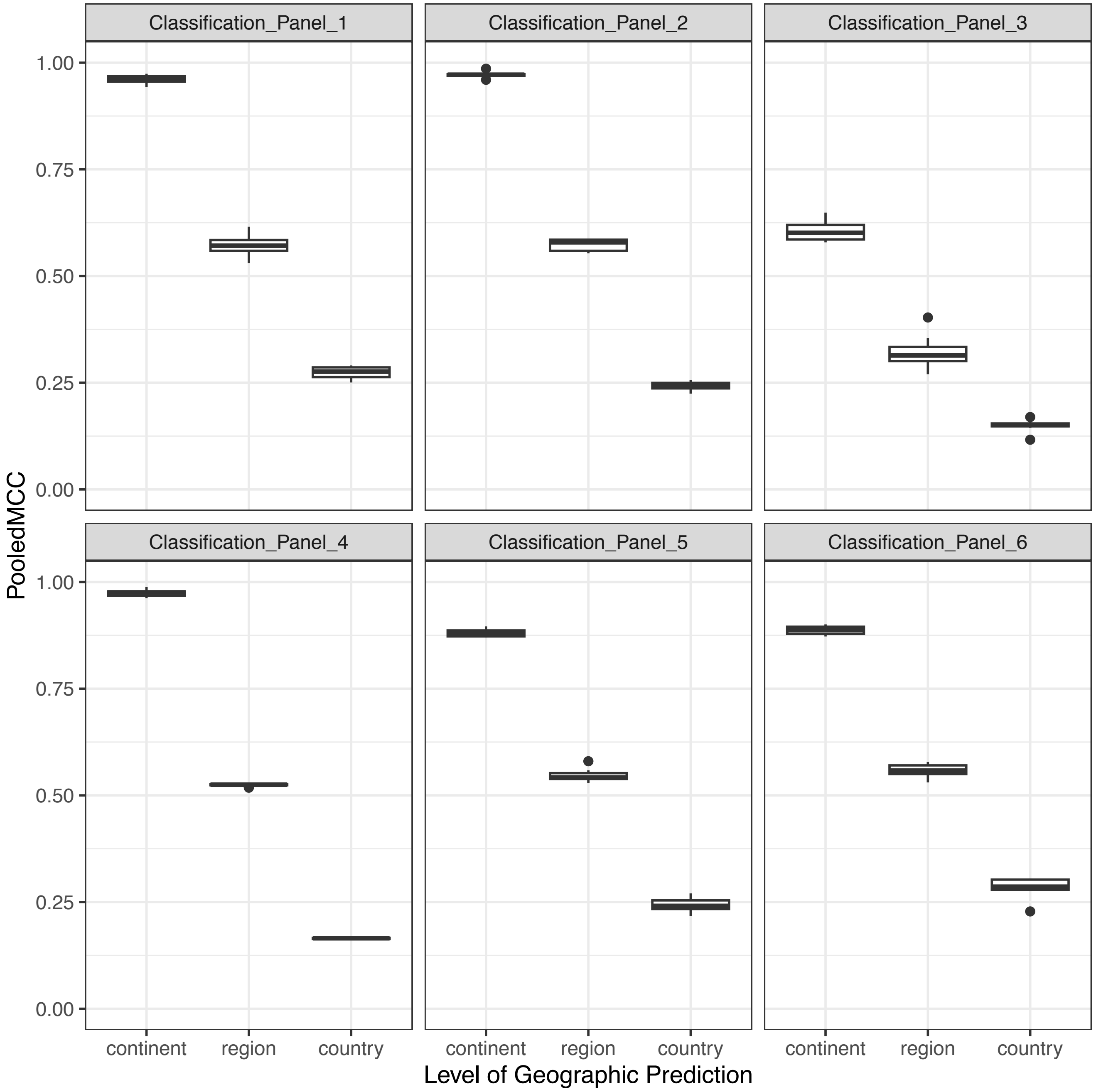

Classification_Panel_1
Classification_Panel_2
Classification_Panel_3
Classification_Panel_4
Classification_Panel_5
Classification_Panel_6
PooledMCC
1.00
0.75
0.50
0.25
0.00
continent
region
country
Level of Geographic Prediction

Figure S2 Legend:

Pooled MCC values for each of the six classification panels at three levels of geographic sensitivity: continent, region, and country. Region level and country level predictions were unreliable with MCC values hovering around 0.5 for region and 0.25 for country level prediction. This compares to >0.9 MCC values for 5 of the 6 classification panels at the continent level. For this reason, region and country level predictions were not used in downstream analysis.

Figure S3

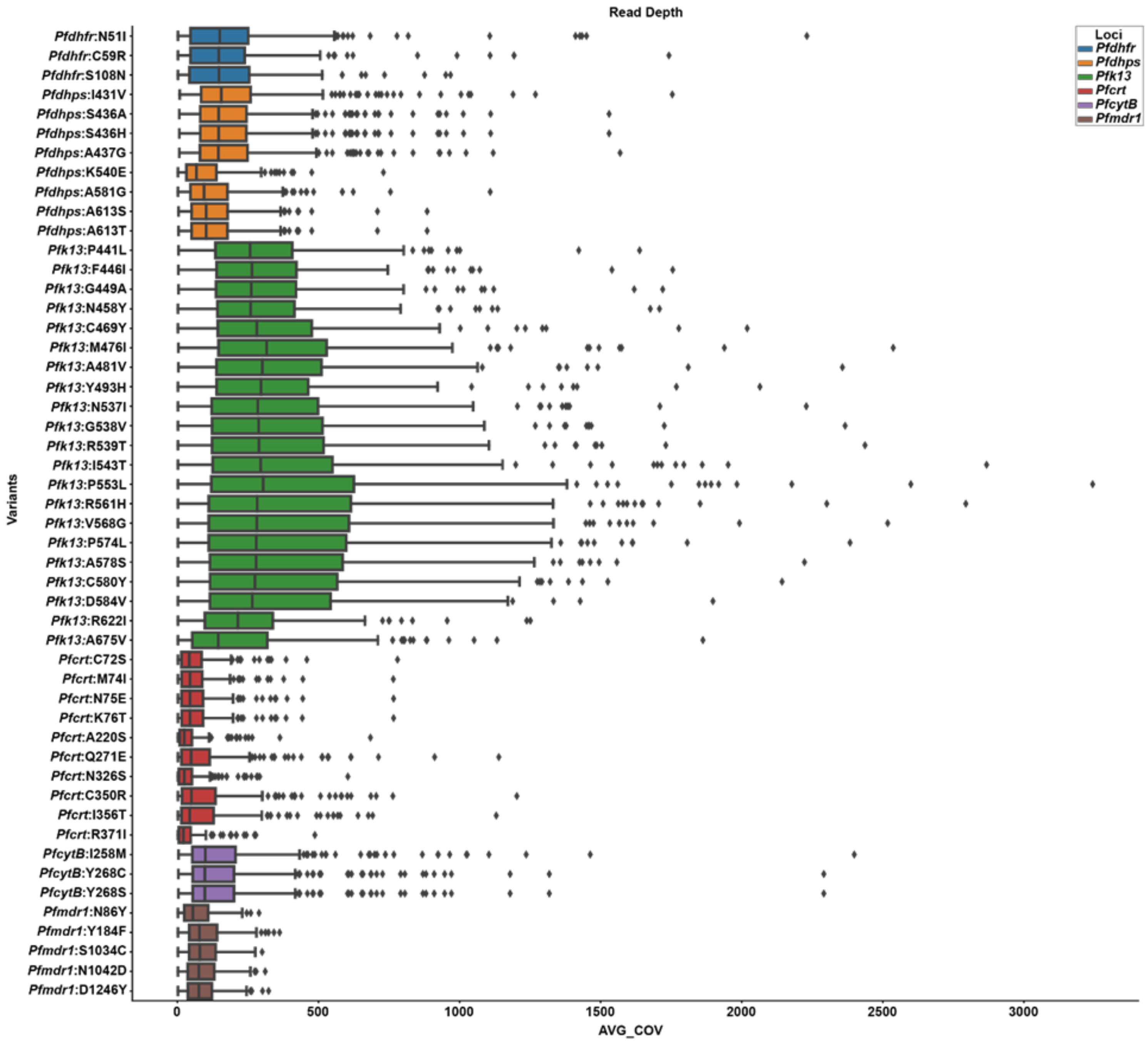

Read Depth
Loci
Pfdhfr
Pfdhps
Pfk13
Pfcrt
PfcytB
Pfmdr1
Variants
Pfdhfr:N51I
Pfdhfr:C59R
Pfdhfr:S108N
Pfdhps:I431V
Pfdhps:S436A
Pfdhps:S436H
Pfdhps:A437G
Pfdhps:K540E
Pfdhps:A581G
Pfdhps:A613S
Pfdhps:A613T
Pfk13:P441L
Pfk13:F446I
Pfk13:G449A
Pfk13:N458Y
Pfk13:C469Y
Pfk13:M476I
Pfk13:A481V
Pfk13:Y493H
Pfk13:N537I
Pfk13:G538V
Pfk13:R539T
Pfk13:I543T
Pfk13:P553L
Pfk13:R561H
Pfk13:V568G
Pfk13:P574L
Pfk13:A578S
Pfk13:C580Y
Pfk13:D584V
Pfk13:R622I
Pfk13:A675V
Pfcrt:C72S
Pfcrt:M74I
Pfcrt:N75E
Pfcrt:K76T
Pfcrt:A220S
Pfcrt:Q271E
Pfcrt:N326S
Pfcrt:C350R
Pfcrt:I356T
Pfcrt:R371I
PfcytB:I258M
PfcytB:Y268C
PfcytB:Y268S
Pfmdr1:N86Y
Pfmdr1:Y184F
Pfmdr1:S1034C
Pfmdr1:N1042D
Pfmdr1:D1246Y
0
500
1000
1500
2000
2500
3000
AVG_COV

Figure S3 Legend:

The read depth coverage for each of the drug resistance-associated polymorphism in six genes. SNP loci are shown on the y axis. Purple, *PfcytB* gene; red, *Pfcrt*; blue, *Pfdhfr*; orange, *Pfdhps*; green, *Pfk13*; brown, *Pfmdr1*. The read depth of coverage is shown on the y axis. The read depth coverage for *Pfcrt* and *Pfmdr1* is lower than the other drug resistance genes. Box plot distribution values: bars = median, lower hinge = 25th percentile, upper hinge = 75th percentile, lower whisker = smallest value no greater than 1.5× interquartile range (IQR) from lower hinge, upper whisker = largest value no greater than 1.5× IQR from upper hinge, and dots = outlier.
